# Video kills the sentiment—Exploring fans’ reception of the video assistant referee in the English premier league using Twitter data

**DOI:** 10.1371/journal.pone.0242728

**Published:** 2020-12-09

**Authors:** Otto Kolbinger, Melanie Knopp

**Affiliations:** Chair of Performance Analysis and Sport Informatics, TUM Department of Sport and Health Sciences, Technical University of Munich, Munich, Germany; Lingnan University, HONG KONG

## Abstract

Evaluative research of technological officiating aids in sports predominantly focuses on the respective technology and the impact on decision accuracy, whereas the impact on stakeholders is neglected. Therefore, the aim of this study was to investigate the immediate impact of the recently introduced Video Assistant Referee, often referred to as VAR, on the sentiment of fans of the English Premier League. We analyzed the content of 643,251 tweets from 129 games, including 94 VAR incidents, using a new variation of a gradient boosting approach to train two tree-based classifiers for text corpora: one classifier to identify tweets related to the VAR and another one to rate a tweet’s sentiment. The results of 10-fold cross-validations showed that our approach, for which we only took a small share of all features to grow each tree, performed better than common approaches (naïve Bayes, support vector machines, random forest and traditional gradient tree boosting) used by other studies for both classification problems. Regarding the impact of the VAR on fans, we found that the average sentiment of tweets related to this technological officiating aid was significantly lower compared to other tweets (-0.64 vs. 0.08; t = 45.5, p < .001). Further, by tracking the mean sentiment of all tweets chronologically for each game, we could display that there is a significant drop of sentiment for tweets posted in the periods after an incident compared to the periods before. A plunge that persisted for 20 minutes on average. Summed up, our results provide evidence that the VAR effects predominantly expressions of negative sentiment on Twitter. This is in line with the results found in previous, questionnaire-based, studies for other technological officiating aids and also consistent with the psychological principle of loss aversion.

## Introduction

79% of the spectators that use a second screen during a live sporting event commit some kind of interaction via social media, for example by posting a comment on Twitter [1]. This widespread use of social media creates very specific, and often very big, data sets that enable researchers to study fan experience, fan behavior, and emotional reactions in new ways. It is possible to track sentiment throughout competitions, or even around them, and therefore, to investigate the influence of certain events [2–4]. A type of event that is new in football and which might have an impact on fans is the use of the Video Assistant Referee (often referred to as VAR) in elite-level football. The VAR was officially introduced by the International Football Association Board (IFAB) in 2018, after several years of pilot studies, off-field tests, and live experiments (e.g. the 2017/2018 season of the German Bundesliga and the Italian Serie A). The IFAB intends to use the VAR to correct “clear and obvious errors” or “serious missed incidents” ([5]: 3) related to (potential) goals, penalties, direct red cards, or mistaken identities.

The only two quantitative studies investigating the VAR focused on its potential impact on various on-field events in different leagues, namely the German Bundesliga, the Italian Serie A, and the Chinese Super League [6, 7]. In both studies, the authors found that the number of offside calls, fouls and yellow cards decreased significantly and that, not related to specific events, the overall duration of games increased slightly. Readers not familiar with this topic might wonder, why there are no studies about usage patterns and the decrease of wrong decisions. Indeed, a majority of the associations using the VAR, provided such studies during the experimental stages and in the aftermath of competitions, e.g. after the FIFA World Cup 2018. Unfortunately, those associations did not provide sufficient information about the study design, which makes it impossible to rate the reliability and validity of the respective results (see [5] as an example). This is unfortunate, as the amount of such reports might have deterred researchers to conduct scientific studies regarding such questions.

In addition, neither such reports nor scientific studies have investigated the influence of the VAR on fans and spectators so far. This is a well-known problem for the evaluation of technological officiating aids (or short: TOA), as Kolbinger and Lames [8] showed in a systematic review. The authors pointed out that the introduction of TOA needs to be treated as interventions in a social setting and therefore, the investigation of stakeholders’ opinions has to play a crucial role in the evaluation process. However, so far, research in this area almost exclusively focused on the influence of such aids on on-field events [9–11], with few exceptions. Winand and Fergusson could show in their study about the goal line technology in football that even if a TOA works smoothly, fans and spectators still express some aversion [12]. In another study about rugby, Stoney and Fletcher [13] showed that fans are generally in favor of the so-called Television Match Official, but perceived a lack of transparency. Both studies used a small triple-digit number of online questionnaires (n = 270 and n = 194 respectively), in which they asked for the general perception of the technological officiating aid, but did not examine the impact of specific events. However, no study has been conducted so far that explores a large number of immediate emotional reactions related to specific incidents involving TOA during live sporting events.

This shortcoming can now be addressed by the use of immediate responses expressed by fans via social media as an indicator of their sentiment. An approach that has already been used successfully to investigate the influence of other events in sports. Lucas et al. [14] showed that tweets during games that were more imbalanced then expected were more negative than tweets in other games. In another study, looking at specific groups of fans, Yu and Wang [15] found that supporters of the US male national team expressed more negative emotions after their team conceded a goal at the 2014 World Cup. Similarly, Fan et al. [16] showed how English football fans showed higher identification with their national team if they were ahead in the score and were more likely to disassociate themselves from the team if it was losing. As it has been done in those studies as well, here we trained a classifier to categorize each tweet’s sentiment automatically but did it in a new way. In more detail, we decided to use a gradient boosting approach to train a tree-based model in a new way. Usually, such approaches use all (or almost all) variables for each tree, whereas we decided to use only a small fraction (10%) of the variables, to better fit the needs for text classification. The reasoning as well as the approach will be described in more detail in our methodology section, in which we also showed how this classifier performed compared to common approaches.

In addition, we developed a model to rate if a tweet was related to the VAR, which is an issue of topic identification. Note that different to, for example goals, not all incidents of interest for us occur on official or reliable sources nor can they be collected otherwise (e.g. by using live video footage). Over 70% of the reviews by the VAR are so-called silent reviews, in which not even the on-field official gets informed about the on-going reviews and far below 10% of the reviews lead to a revision of the on-field decision (see [17] for a detailed summary for such numbers in the German Bundesliga). However, incidents in which the VAR did not intervene can cause emotional reactions as well, especially if spectators do not agree with the VAR’s non-intervention. Adopting a topic detection algorithm helped us to detect such incidents as well. Using Twitter data to detect real world events has not been applied in peer-reviewed literature of sports science so far, but has successfully been adopted in other fields, e.g. to detect earthquakes [18] or to explore abnormal stock price returns [19].

To sum up, this paper provides two kinds of contributions. First, we apply a new machine learning approach for automated text classifications in a way that has not been adopted for such kind of problem before. The complete approach, including data preprocessing, and its evaluation are described in detail in the first part of the methodological section. In the second part of this section we display how we synthesize the information retrieved from sentiment analysis and topic detection to evaluate the immediate impact of the Video Assistant Referee on the sentiment of football fans. Therefore, we do not just explore the difference in sentiment for tweets related to this topic compared to other tweets, but also how specific VAR incidents influenced the overall sentiment during football games. As this second contribution is the main purpose of this study, the results and the discussion of this paper will focus on the findings regarding the VAR’s impact. In our concluding section we will try to point out how these findings can serve sports associations and how our methodological approach can contribute to the knowledge base regarding automated text classification.

## Methodology

### Sample

#### Data & materials

Using the official Twitter API, we collected all the tweets during 129 matches of the 2019/2020 Premier League (EPL) season, which were tagged with the official game hashtag (the included games are listed in S1 Table). This official hashtag and chronological information, like kick off time, were retrieved from the official website of the league (www.premierleague.com). Data collection for each game was started at the minute the game officially started and stopped after 120 minutes for each game, to ensure that tweets during the whole match were extracted. Our final sample consists of 643,251 tweets, a mean of 4,986.4 tweets per game, with the number of tweets per game ranging from 40,127 to 284 (variation coefficient of 1.24). The number of tweets for each game can be found in the list of games (S1 Table).

#### Training data

As training data, a subsample of 4,583 tweets was drawn from the overall sample, including tweets from 24 games. To make sure that this data set covered the most common VAR incidents, the games were not drawn randomly but selected based on the occurrence of certain VAR incidents. Again, the VAR can be used to check goals, penalties, red cards, and if the right player got booked (so-called *mistaken identity*). The latter situation is very rare and did not appear in our sample. For the other three incidents, we selected games including different variations of those, as for example for a goal, the VAR can be used to confirm or overturn the initial decision due to a possible handball, foul, or offside call. The list of games, including information about the VAR incident can be found in the supporting information (S2 Table).

For each of the 24 games, we randomly drew a subsample of a maximum of 100 tweets during the VAR incident (using a three-minute window starting at the minute the VAR intervened) and a random sample of the same size during the rest of the game. For each of these tweets, one of the two authors rated whether the tweet was related to the Video Assistant Referee (1 if true, 0 if not) and classified the overall sentiment of the tweet as either positive (1), neutral (0) or negative (-1). This led to the following composition of our training data: 31.1% of the 4,583 tweets were labelled as related to the Video Assistant Referee. Regarding sentiment, 25.5% were rated as an expression of positive sentiment, whereas 41.1% were rated as negative.

The tweets for one game were rated by both observers to check the inter-annotator agreement. Cohen’s Kappa for the topic detection was 0.99, Krippendorff’s Alpha for the rating of the sentiment reached 0.88. Krippendorff’s Alpha is used for the sentiment classification throughout this paper, instead of Cohen’s Kappa, as it takes the ordering of categories into account and therefore, is more appropriate for this kind of classification problem [20].

### Data preprocessing

Before the Twitter data was used, several steps of preprocessing were performed. Besides standard text mining procedures (removing punctuation, converting to lower text, removing numbers, stemming), a customized list of words was excluded if they occurred in a tweet, including team and player names as well as respective variations of those and team specific hashtags (e.g. #lfc or #mufc). See S3 Table for this list of customized stop words. We did not exclude so-called general stop words, because these words can associate with expressions of sentiment (e.g.: “not”, “should”). In addition, an emoji dictionary was created to transform emojis into text that can be processed by text classification algorithms. The emoji dictionary as well as the function used to transform the emojis is stored here: https://github.com/PostdOK/preprocessing_emojis.

As we wanted to build our feature set based on unigrams for all the classification models, we then converted our data set into a conventional document-term-matrix. No further filtering or weighting schemes were applied.

### Text classification and analysis

#### Models

Several supervised machine learning algorithms are suitable to train topic detection or sentiment classification models for short text corpora. For both problems, we decided to use a gradient boosting approach to train a tree-based model, as such approaches showed promising results in studies working on similar problems [16]. Boosting approaches usually use a bootstrap sample for each tree, including the information of almost all (or even all) the variables. However, as automated text classification models get trained based on a myriad of variables we assume that it might be beneficial to combine the information of a lot of decision trees that only include a small fraction of the variables (10%) to avoid overfitting. To our best knowledge, such an approach has not been published before. To verify our methodological decision, we also ran “traditional” boosting models using the information of all variables for each tree. Further, we adopted three further classification models: a naïve Bayes classifier, a support vector machine, and the bagging-based counterpart to our approach, a random forest. All models used exclusively unigrams as feature set.

To evaluate the performance of the different classification models, we ran standard 10-fold cross-validations for all models. In more detail, we split the training data of 4,583 tweets into ten almost equally-sized folds (three folds of 459 tweets and seven folds of 458 tweets). For each model, we then estimated the performance ten times, using each fold once as test set and the remaining nine folds as training set. The performance measures of these 10-fold cross-validations for both classification problems are displayed in Table 1. Our approach performed slightly better than the traditional boosting approach and the other models for both, sentiment classification and topic detection (VAR). For the latter, all the three approaches based on decision trees reached an *accuracy* value of 94% and also did not differ much concerning the *F-Score* (random forest: 0.954, traditional gradient boosting: 0.954, new gradient boosting: 0.956). The support vector machine performed slightly worse (*accuracy*: 91.5%; *F-Score*: 0.938), whereas the naïve Bayes classifier could not reach such a high level due to a very high number of false positives, as it is displayed by the *precision* value of just 71.0% (*accuracy*: 79.1%; *F-Score*: 0.824).

**Table 1:** Performance of the five adopted classifiers for topic detection and sentiment classification.

Classifier	Metric	VAR—detection	Sentiment		
			Negative	Neutral	Positive
**New gradient boosting–decision trees**	Overall Accuracy	0.94		0.71	
	Krippendorff’s Alpha	-		0.53	
	F-Score*	0.96		0.68	
	Precision	0.97	0.79	0.72	0.57
	Recall	0.94	0.74	0.72	0.62
**Naïve Bayes**	Overall Accuracy	0.79		0.69	
	Krippendorff’s Alpha	-		0.50	
	F-Score*	0.82		0.67	
	Precision	0.71	0.86	0.53	0.61
	Recall	0.98	0.69	0.88	0.55
**Support Vector Machine**	Overall Accuracy	0.92		0.69	
	Krippendorff’s Alpha	-		0.51	
	F-Score*	0.94		0.66	
	Precision	0.94	0.76	0.70	0.56
	Recall	0.94	0.74	0.70	0.59
**Random Forest**	Overall Accuracy	0.94		0.66	
	Krippendorff’s Alpha	-		0.43	
	F-Score*	0.95		0.60	
	Precision	0.96	0.81	0.68	0.39
	Recall	0.95	0.66	0.68	0.60
**“Traditional” gradient boosting–decision trees**	Overall Accuracy	0.94		0.69	
	Krippendorff’s Alpha	-		0.50	
	F-Score*	0.95		0.65	
	Precision	0.97	0.73	0.70	0.52
	Recall	0.95	0.75	0.69	0.60

* We calculated the macro-averaged F-Score for sentiment classification.

For sentiment classification, our approach reached the highest values for *accuracy* (70.8%), the metric most often referred to as performance measure, as well as for the macro-averaged *F-Score* (0.678) and Krippendorff’s *Alpha* (0.530), which we considered as more important measures for sentiment classification, as these values take the ordering of the categories into account [21, 22]. All models performed better for negative sentiment than for positive sentiment. It needs to also be noted that all models performed on a similar level, except the random forest classifier.

Consequently, we decided to classify all tweets of our sample based on the classifier developed by our new gradient boosting approach. Compared to other studies dealing with the same problem–automated classification of sentiment for three classes of tweets in English–we reached equivalent performance values. Using a much bigger training data set of 103,262 tweets, Ranco et al. [19] accomplished an *accuracy* value of 76.1%, but only a macro-averaged *F-Score* of only 50.8% (Krippendorff’s Alpha was not displayed). Mozetic et al. [20] reported an *accuracy* of 63.9%, a Krippendorff’s *Alpha* of 51.6%, and a macro-averaged *F-Score* of 0.530 (n = 103,034 tweets).

#### Identification of VAR incidents

**Fig 1:**
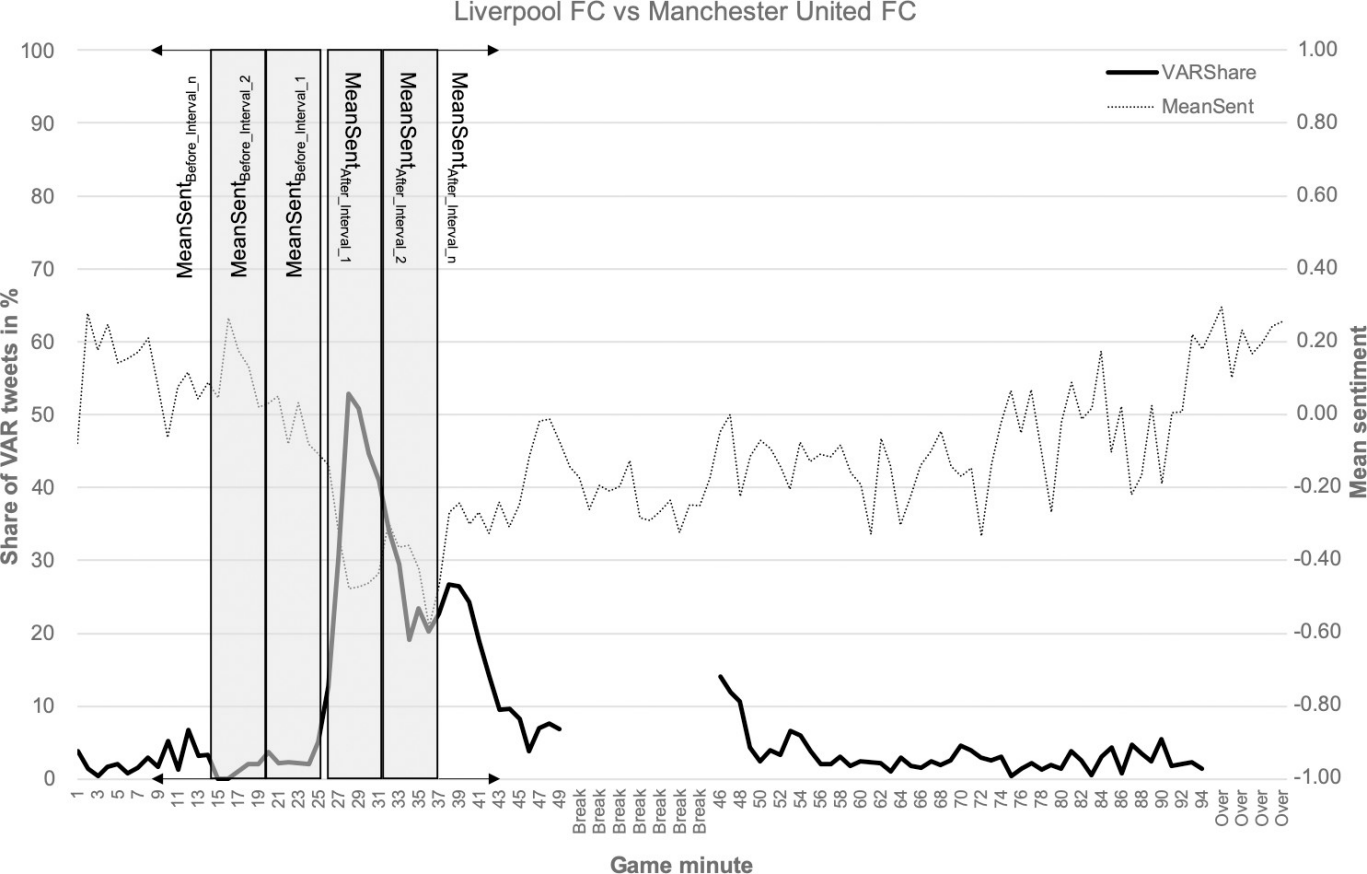
Share of VAR tweets and mean sentiment for each minute of Liverpool FC vs. Manchester United FC. The grey shadowed boxes display the procedure described in statistical analysis.

To be identified as a VAR incident an increase of tweets related to the VAR had to meet several conditions. As it can be seen in Fig 1, erroneously labelled tweets related to the VAR or random tweets not related to a specific incident, can lead to a certain level of noise in our data. To deal with this background noise, we defined two conditions that had to be met by a VAR incident. First, the increase in the number of tweets related to the VAR had to be greater than the sum of such tweets in the previous five minutes. Second, as due to the nature of text classifiers such an increase could be affected solely by an increase in the number of tweets, over 25% of the tweets had to be related to the VAR. Using such a high threshold should also serve the purpose of identifying incidents in which the VAR was mentioned by a considerable share of people tweeting about this game.

Using this approach, we identified 95 incidents, which were double-checked manually by one author. Only one of these incidents was not related to the VAR, but rather to the overall performance of the referee, wherefore this incident was excluded from further analysis. Of the remaining 94 incidents, 65 were listed in the live match commentary of the official homepage of the English Premier League. 28 incidents were related to cases in which people complained about the absence of an intervention of the VAR. As mentioned above, for those there is no official information whether or not the video assistant checked the respective incident. In addition, not even all interventions were reported by the official live match commentary on www.premierleague.com, for example, an overturned goal due to a handball in a match between Tottenham Hotspur FC and AFC Bournemouth. S4 Table is a list of the detected incidents.

#### Statistical analysis

To investigate the influence of the VAR on the sentiment of football fans expressing themselves via Twitter, we ran several statistical analyses. Without further preprocessing, we first investigated the relationship between the share of different sentiments and the number of tweets for the whole sample as well as for the subset of tweets related to the VAR. Second, a paired t-test was run to compare the sentiment of VAR tweets to the other tweets, based on the mean sentiment value of each game for both categories. Third, we split each game into 24 five-minute intervals and calculated the mean sentiment for each interval with more than 25 tweets (reasoning: this way, no additional tweet can change the mean sentiment by more than 5%). We then looked if the share of tweets related to the VAR was over- or underrepresented for each vigintile using the Kolmogorov-Smirnov test and a similar distribution over all vigintiles as the null hypothesis.

In addition, we used VAR incidents identified by our method described above and extracted the mean sentiment over five-minute windows (*MeanSent*) before and after each incident. In more detail, if the incident was identified for minute t, *MeanSent*_*Before_Interval_1*_ started at t-6 and stopped at t-2, *MeanSent*_*Before_Interval_2*_ started at t-11 and stopped at t-7, and so on. Minute t-1 was excluded to avoid interferences with the VAR incident. The *MeanSent*_*After_Interval_1*_ window included the sentiment values from the minutes t to t+4, the *MeanSent*_*After_Interval_2*_ window the values from t+5 to t+9, and so on. This procedure is also displayed in Fig 1. If another VAR incident happened during the same game, only the mean sentiment for intervals until this point were calculated (and vice versa if an incident occurred before in the same game). In addition, the mean sentiment was only calculated if the number of tweets was at least 25 for any considered period.

This approach enabled us to perform several further analyses. First, the last interval before an incident (*MeanSent*_*Before_Interval_1*_) was compared to all the preceding intervals to investigate if there is a stable sentiment pattern despite the intra- and inter-game variation. Second, the comparison of intervals right before and after the incident provides insights into the immediate impact of a VAR incident on fans’ sentiment. Third, tracking the sentiment for the remainder of the game after such incidents, allowed us to investigate if and when the sentiment reached the pre-incident level again.

All those comparisons were performed via paired t-tests. Games did not provide mean sentiment values for the same number and kind of windows, as the VAR incidents happened at different minutes in the game. Thus, we decided to only use those windows that included at least one third of the games, which applied for nine windows before and nine windows after the incident.

### Programming

All the steps described above were performed via R utilizing the following packages: tweets were collected using *rtweet* and prepared for the different automated classification approaches using *tm*, *dplyr*, *quanteda*, and *tidytext*. The *quanteda*.*textmodels* package was used to train the naïve Bayes classifier, *caret* for the support vector machine, *randomForest* for the Random Forest, and *xgboost* for the different variations of the gradient boosting approaches. For our analysis, we relied on different functions from *stats*, *irr*, and *cocor*.

## Results

### Sentiment patterns

**Fig 2:**
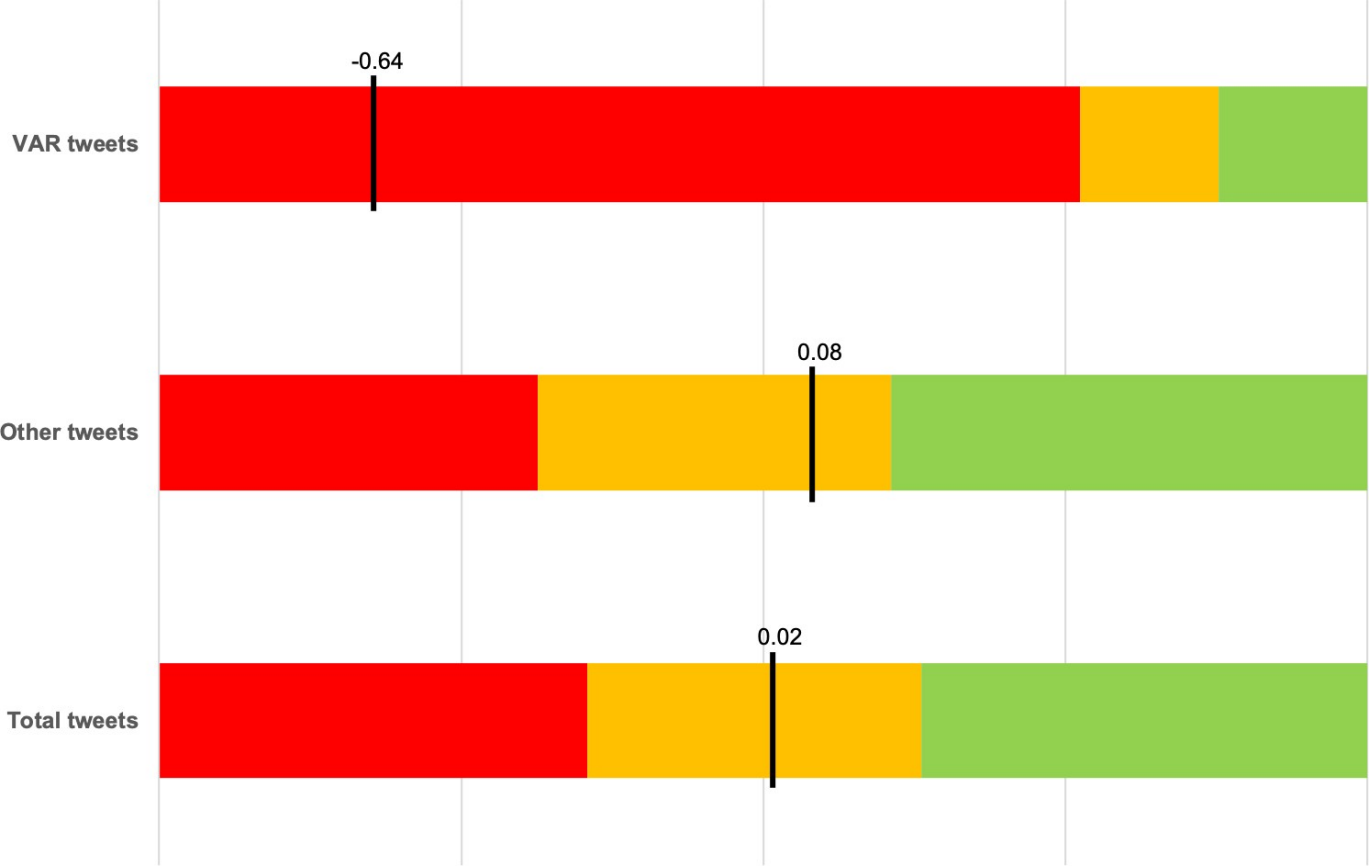
Sentiment distribution of all tweets (*Total tweets*), tweets related to the VAR (*VAR tweets*), and tweets not related to the VAR (*Other tweets*). Negative tweets are denoted by red, neutral by orange, and positive by green color. The black line denotes the average sentiment score.

As it is displayed in Fig 2, far more tweets were labelled as positive (36.9%) or negative (35.4%) than neutral (27.7%). The average sentiment of all tweets of our sample was 0.02. Looking on a game to game basis, the total number of tweets showed a medium to strong positive linear correlation with the share of positive tweets (r = .324, t = 3.86, p < .001), as well as with the share of negative tweets (r = .406, t = 5.01, p < .001), with no significant differences between the two correlation coefficients (z = 0.71, p = .475). In contrast, the share of neutral tweets has a strong negative linear correlation with the total number of tweets (r = -.529, t = -7.03, p < .001), differing significantly from the other categories (vs. positive: z = 5.98, p < .001; vs negative: z = 6.12, p < .001).

A total of 58,264 tweets and therefore, 9.1% of the overall tweets were related to the Video Assistant Referee. Those tweets showed a different sentiment distribution, with the vast majority of tweets labelled as negative (76.2%) and an almost equal share labelled as positive or neutral (12.3% and 11.4%). Consequently, there was a medium to strong positive linear correlation between the number of tweets related to the VAR and the negative share of overall tweets in a game (r = .480, t = 6.17, p < .001). There was no correlation for neither the positive share (r = -.030, t = -0.34, p = 0.738) nor the neutral share (r = -.376, t = -4.57, p < .001), with both correlation coefficients differing significantly from the one of the negative share (vs. positive: z = 4.32, p < .001; vs. neutral: z = 5.49, p < .001). The average sentiment of tweets related to the VAR was -0.64, which was significantly lower than the average of 0.08 for the other tweets (t = 45.5, p < .001).

**Fig 3:**
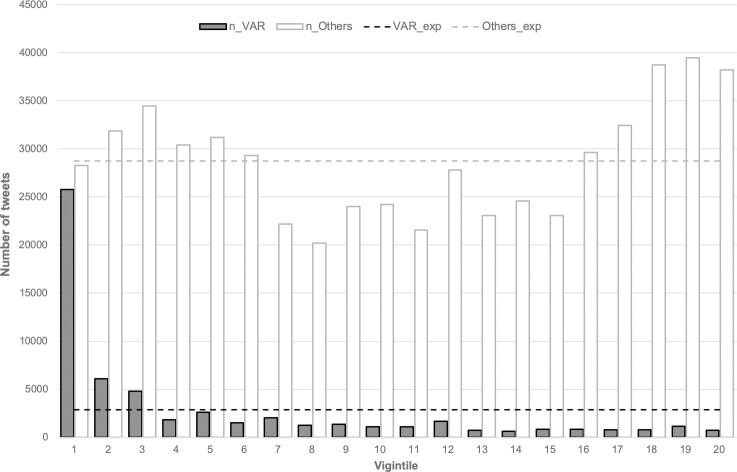
Number of tweets related and not related to the VAR per vigintile. The dashed line in dark grey displays the expected number of VAR tweets, the one in light grey illustrates the expected number of tweets not related to the VAR for each vigintile.

To further illustrate the influence of the VAR on the sentiment of fans, we calculated the mean sentiment for each five-minute period of each game as described above. We then calculated the quantile-rank of the mean sentiment for each such period and checked if the total number of VAR tweets was distributed evenly over all twenty vigintiles. As it can be seen in Fig 3, the number of tweets related to the VAR exceeded the expected number of such tweets for the three vigintiles with the lowest average sentiment values. For the remaining vigintiles, the expected number of VAR tweets was not reached anymore and for six out of the seven most positive vigintiles not even one third was matched. A Kolmogorov-Smirnov test confirmed that this distribution is significantly different from an even one (d = 0.85, p < .001). It also differed significantly from the respective distribution of the number of tweets not related to the VAR, which was rather U-shaped, as it can be seen in Fig 3 as well (d = 0.95, p < .001).

### Sentiment response to VAR incidents

**Fig 4:**
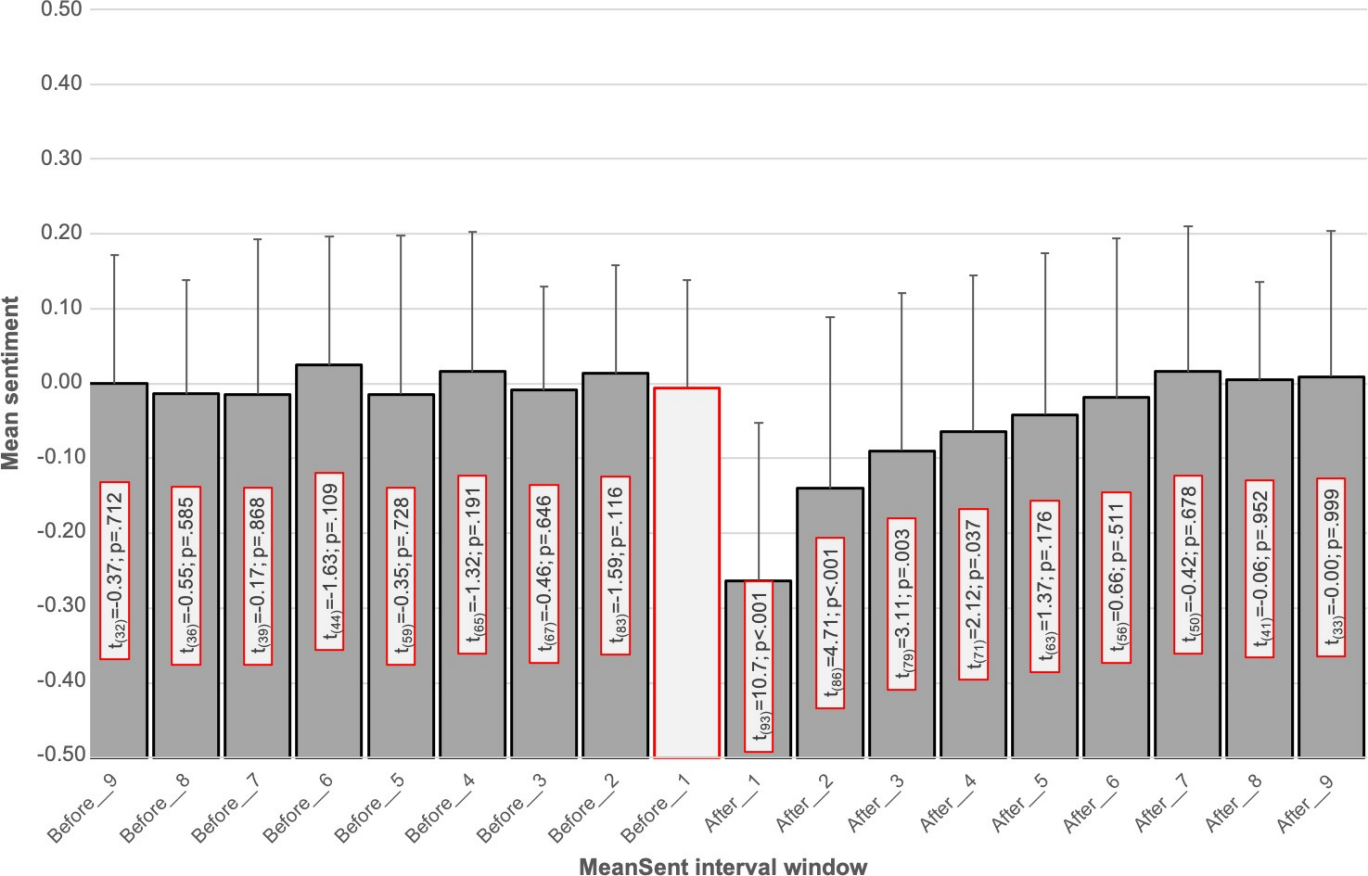
Mean sentiment for the nine five-minute windows before and after VAR incidents. The results of the paired t-test for the comparison of each window with the immediate window before the incident (MeanSent_Interval_Before_1_, grey bar with red border) are displayed in the respective bars.

To investigate the exact influence of specific VAR incidents we compared the mean sentiment of different time intervals before and after 94 incidents. The results are displayed in Fig 4. To investigate the sentiment variation before VAR incidents, we compared the mean sentiment of the interval MeanSent_Before_Interval_1_ to all other intervals in advance of the incidents. As can be seen in Fig 4, this interval did not differ significantly from any previous interval.

As soon as a VAR incident was identified, the mean sentiment dropped significantly: for the immediate reaction, comparing *MeanSent*_*Before_Interval_1*_ and *MeanSent*_*After_Interval_1*_, we found a significant mean difference of 0.26 (t = 10.7, p < .001). The mean sentiment did not recover and still showed a significant decrease for the next three intervals *MeanSent*_*After_Interval_2*_ (mean of the differences: 0.13, t = 4.71, p < .001), *MeanSent*_*After_Interval_3*_ (mean of the differences: 0.09, t = 3.11, p = .003) and *MeanSent*_*After_Interval_4*_ (mean of the differences: 0.06, t = 2.12, p = .037).

## Discussion

The widespread use of social media opens new opportunities for researchers to study immediate responses and sentiment during–or related to–events. An opportunity that has already been embraced by researchers from several different research areas [23]. For this work, we used tweets posted during 129 matches of the English Premier League to investigate the impact of the recently introduced Video Assistant Referee on spectators’ sentiment. Before we discuss the results regarding this TOA, it is useful to point at some interesting findings regarding the overall sentiment patterns first.

Lucas et al. [14] found that more tweets being posted related to one game usually came along with a higher share of negative sentiment expressions, but not with a higher share of positive sentiment expressions. For our tweets, especially excluding tweets related to the VAR, we also found a medium to strong positive correlation for this share of positive labelled tweets. Based on the insufficient evaluation of their method, it is not possible to analyze if the respective automated classifier applied by Lucas et al. can explain this difference. One explanation could be that those authors only used English tweets to track the sentiment, but did so for almost all games at the 2014 FIFA World Cup, with most of the games not including teams from anglophone countries. However, Yu and Wang [15] found that if fans tweeted about games for which they (supposedly) had no rooting interest, they expressed positive emotions more frequently than negative ones. In accordance with these findings, the patterns for the small number of games investigated by Fan et al. [16] showed a similar relationship between the number of tweets and the share of positive sentiment. This also applies to findings from questionnaire-based surveys that show that people post on social media to express their emotions in general, but not to express particularly negative or positive emotions [24].

Interestingly, we only found the same pattern as Lucas et al. [14] for a subset of our tweets: tweets related to the video assistant referee. An increase in the number of such tweets went along with an increase in the share of negative tweets, but not with the share of positive or neutral ones. Further analysis confirmed that tweets related to the VAR are significantly more negative (mean sentiment: -0.64) than other tweets (mean sentiment: 0.08). This is not just an impressive difference, considering that the mean sentiment can only range between values from -1 to 1, but also looking at the results from other studies. Fan et al. [16] only reported a sentiment difference of 0.26 for fans of the English national team if they were trailing compared to winning for games at the World Cup, the most important tournament in football (see i.a. [25]).

Besides the overall sentiment characteristics of tweets related to the VAR, we also looked at the immediate impact of VAR incidents. Comparing our results to those of previous findings, one has to keep in mind that our samples for each game included responses of fans from both teams as well as neutral spectators. Each change (or no-change) of an on-field decision does not just result in a disadvantage for one team, but also benefits the other team, and all the respective reactions are displayed in our results (e.g. the above-mentioned difference reported by Fan et al. [16] only expressed the sentiment swing for fans of one side). Still, we found that the mean sentiment for all tweets before and after an incident dropped by 0.26 and remained on a significantly lower level for the next 20 minutes.

Reasons to explain those predominately negative reactions towards the VAR can be found in practice as well as in theory. First, even Michael Riley, the Premier’s League head of officiating, admitted that the VAR was not consistently applied the way it should be. He even admitted that several correct on-field decisions were overruled [26]. Stakeholders that are responsible for interventions in a social setting are interested in selling these as a success, often irrespective of its actual merit. That such stakeholders criticize their interventions publicly and therefore, their decision to some extent, is highly unusual [27].

Second, especially the high share of negative expressions after VAR incidents needs to take loss aversion into account [28]: humans weigh losses higher than they weigh wins. In addition, the loss of satisfaction also depends on the reference level. Consequently, fans that are, for example, on the wrong side of a disallowed goal are likely to perceive relatively more negative emotions than fans of the benefiting team perceive positive ones.

Third, the few studies investigating the impact of technological officiating aids on fans and spectators showed that even if a TOA worked quite well, it might not be appreciated by everybody. Looking at a similar intervention in rugby, Stoney and Fletcher [13] described that for the vast majority of fans the use of the Television Match Official does interfere with their fan experience, inter alia because they do not get proper information about the decisions made. Winand and Fergusson [12] showed for another TOA in football, the goal line technology, that only two thirds of the fans were in favor of this TOA, even though over 90% believed it is accurate. In addition, the participants in their study tended to dislike the idea of implementing (further) technological officiating aids. To sum up, the respective football associations, in our case the English Premier League, need to be aware that these studies provide strong indications that the video assistant referee interferes with fan experience, which is in line with our findings.

To further verify our loss aversion argument, future studies could try to inspect the sentiment of the involved fan bases separately. That we did not do so can be seen as a limitation of this study. However, one has to respect that approaches used by other studies, were not feasible for this study (like using the tweets’ language) or were not considered appropriate (like the location stated in the user’s bio, which can be changed at will). As previous studies found that a decent share of tweets included some kind of expression of sympathy for one team, using such information could be a promising approach [29]. However, people turn frequently to Twitter to discuss on-going events during live sporting contests, especially if they perceive themselves as experts [30], irrespective of their rooting interests. Consequently, only using tweets of the involved fanbases can lead to a significant decrease of tweets that can be used for further analyses.

Another limitation of this study is that we only looked at games of the EPL. Thus, possible differences compared to other leagues need to be considered if one wants to draw general conclusions. As an example, the EPL has professional referees–the German Bundesliga has not. On the other hand, the German Bundesliga introduced the VAR already in the 2017/18 season, whereas the EPL waited until 2019/20. Further, governing bodies can pursue different strategies of communicating decisions or changes in the procedures. Nevertheless, all competitions use the same underlying rules and regulations for the VAR.

## Conclusion

With this work, we present evidence that the recently introduced Video Assistant Referee has a negative impact on the sentiment of spectators of the English Premier League that post on social media. We did so by developing a new approach for automated text classification, a variation of a gradient boosting algorithm, which we consider as another important contribution of this paper. As the results of 10-fold cross-validations revealed, this approach outperformed other common approaches for topic detection and sentiment classification for our data. We are aware that the quality of sentiment classifiers depend on the topic, which means that a classifier trained on tweets related to one topic, can perform worse on other topics [31]. This makes it hard to compare the performance measures to those from other papers. However, especially considering the high values for Krippendorff’s Alpha and the macro-averaged F-Score, two metrics especially suited to measure the performance of sentiment classification, we want to encourage researchers to test this approach for further data sets related to other topics or even for datasets including several different topics.

Regarding the Video Assistant Referee, several theoretical and practical conclusions can be derived from our study. So far, neither scientific studies nor practical evaluations investigated the immediate impact of a technological officiating aid on stakeholder groups. We did not just show how this can be accomplished using immediate responses expressed by fans via social media, but also demonstrated the merit of this information. Our study revealed that the VAR has a substantial negative impact on fan experience, which needs to be taken into account by the governing bodies in football.

It needs to be evaluated how this negative impact can be reduced, for which we want to suggest two possible paths. First the football associations should try to ensure as much transparency as possible throughout VAR uses. Stoney and Fletcher [13] highlighted how important it is for fans to get proper information about the decision-making process of replay reviews. To provide this transparency, the associations could stream the communication between the referee and the video assistant (as it is done in field hockey) or equip referees with proper technology to announce information about the review process in the stadium (as it is done in the NFL).

Another way to enhance the fan experience in relation to the VAR could be the implementation of a challenge system in which the competing parties are responsible to initiate the review process by appealing against the on-field call. In football, the IFAB decided right away to implement a different system in which the video assistant is responsible to initiate reviews by double-checking every possible incident. This did not just lead to a nontransparent system but also put the burden for the merit of this TOA on the shoulders of the referees exclusively. Other sports use challenge systems for different TOA, e.g tennis or baseball, which takes some pressure away from the referees, as the athletes or coaches themselves have to (immediately) select the scenes that should be reviewed. The strategy to use such challenges, as athletes or teams usually have a limited number of those, has become a much sought-after research topic, which could be an indication of how challenge systems improve fan experience [32–34].

Irrespective of whether or not the suggested paths are seen as proper solutions, our findings show that the status quo cannot be considered as satisfying and football’s governing bodies need to improve the current system.

## Supporting information

S1 Table List of all games of the sample, including the number of extracted tweets for each. (CSV)

S2 Table Games and VAR incidents included in the training data. (CSV)

S3 Table List of customized stop words. (CSV)

S4 Table Detected VAR Incidents. “MinuteSinceKickOff” displays the minute in which the VAR incident was detected by our algorithm.
(CSV)

S5 Table Data for all 643,251 tweets. Each line includes the following information: game in which the tweet appeared (“Game”), the number of minutes passed since kickoff (“MinuteSinceKickOff”), whether the tweet is related to the VAR (“VAR”) and the sentiment (“Sentiment”). “NewID” is an ID assigned by us, which allows us to relate this information to the original tweet.
(CSV)

S6 Table Definition of the performance measures used to evaluate the classifiers. (DOCX)

## References

[1] Cunningham NR, Eastin MS. Second screen and sports: a structural investigation into team identification and efficacy. Communication & Sport. 2017;5(3): 288–310. 10.1177/2F2167479515610152

[2] Bhatia S, Mellers B, Walasek L. Affective responses to uncertain real-world outcomes: sentiment change on Twitter. PLoS ONE. 2019;14(2): e0212489 10.1371/journal.pone.0212489 30811456 PMC6392292

[3] Feddersen A, Humphreys BR, Soebbing BP. Sentiment bias and asset prices: evidence from sports betting markets and social media. Econ Inq. 2017;55(2): 1119–1129. 10.1111/ecin.12404

[4] Frederick E, Burch L, Blaszka M. A shift in set: examining the presence of agenda setting on Twitter during the 2012 London Olympics. Communication & Sport. 2015;3(3): 312–333. 10.1177/2167479513508393

[5] The International Football Association Board [Internet]. Zurich, Switzerland: The International Football Association Board; 2018 Mar 3 [cited 2020 June 14]. Available from: http://static-3eb8.kxcdn.com/documents/648/071316_030318_AGM_Media_Package_Final.pdf

[6] Lago-Peñas C, Rey E, Kalén A. How does Video Assistant Referee (VAR) modify the game in elite soccer? Int J Perform Anal Sport. 2019;19(4): 646–653. 10.1080/24748668.2019.1646521

[7] Han B, Chen Q, Lago-Peñas C, Wang C, Lui T. The influence of the Video Assistant Referee on the Chinese Super League. Int J Sports Sci Coach. Epub 2020 June 29. 10.1177/1747954120938984

[8] Kolbinger O, Lames M. Scientific approaches to Technological Officiating Aids in game sports. Current Issues in Sport Science. 2017;2(1): 10.15203/CISS_2017.001

[9] Abramitzky R, Einav L, Kolkowitz S, Mill R. On the optimality of line call challenges in professional tennis. Int Econ Rev. 2012;53(3): 939–964. 10.1111/j.1468-2354.2012.00706.x

[10] Clarke RS, Norman MJ. Optimal challenges in tennis. J Oper Res Soc. 2012;63(12): 1765–1772. 10.1057/jors.2011.147

[11] Kolbinger O, Link D. The use of vanishing spray reduces the extent of rule violations in soccer. SpringerPlus. 2016;5(1): 1572–1572. 10.1186/s40064-016-3274-2 27652145 PMC5023644

[12] Winand M, Fergusson C. More decision-aid technology in sport? An analysis of football supporters’ perceptions on goal-line technology. Soccer Soc. 2018;19(7): 966–985. 10.1080/14660970.2016.1267629

[13] Stoney E, Fletcher T. “Are fans in the stands an afterthought?”: sports events, decision-aid technologies, and the Television Match Official in Rugby Union. Communication & Sport. Epub 2020 Feb 7. 10.1177/2167479520903405

[14] Lucas GM, Gratch J, Malandrakis N, Szablowski E, Fessler E, & Nichols J. GOAALLL!: Using sentiment in the world cup to explore theories of emotion. Image Vis Comput. 2017;65: 58–65. 10.1016/j.imavis.2017.01.006

[15] Yu Y, Wang X. World Cup 2014 in the Twitter World: A big data analysis of sentiments in U.S. sports fans’ tweets. Comput Human Behav. 2015;48: 392–400. 10.1016/j.chb.2015.01.075

[16] Fan M, Billings A, Zhu X, Yu P. Twitter-Based BIRGing: big data analysis of English national team fans during the 2018 FIFA World Cup. Communication & Sport. 2020;8(3): 317–345. 10.1177/2167479519834348

[17] Kolbinger O. VAR experiments in the Bundesliga In: Armenteros M, Benítez AJ, Betancor MA, editors. The use of video technologies in refereeing football and other sports. New York, NY: Routledge; 2020 pp. 228–245.

[18] Earle P, Guy M, Buckmaster R, Ostrum C, Horvath S, Vaughan A. OMG Earthquake! Can Twitter improve earthquake response?. Seismological Research Letters. 2010;81(2): 246–251. 10.1785/gssrl.81.2.246

[19] Ranco G, Aleksovski D, Caldarelli G, Grčar M, Mozetič I. The effects of Twitter sentiment on stock price returns. PLoS ONE. 2015;10(9): e0138441 10.1371/journal.pone.0138441 26390434 PMC4577113

[20] Mozetič I, Grčar M, Smailović J. Multilingual Twitter sentiment classification: the role of human annotators. PLoS ONE. 2016;11(5): e0155036 10.1371/journal.pone.0155036 27149621 PMC4858191

[21] Kiritchenko S, Zhu X, Mohammad SM. Sentiment analysis of short informal texts. J Artif Intell Res. 2014;50: 723–762. 10.1613/jair.4272

[22] Mozetič I, Torgo L, Cerqueira V, Smailović J. How to evaluate sentiment classifiers for Twitter time-ordered data? PLoS ONE. 2018;13(3): e0194317 10.1371/journal.pone.0194317 29534112 PMC5849349

[23] Zimmer M, Proferes NJ. A topology of Twitter research: disciplines, methods, and ethics. Aslib Journal of Information Management. 2014;66(3): 250–261. 10.1108/AJIM-09-2013-0083

[24] Wang X. Applying the integrative model of behavioral prediction and attitude functions in the context of social media use while viewing mediated sports. Comput Human Behav. 2013;29(4): 1538–1545. 10.1016/j.chb.2013.01.031

[25] Crabbe T. Fishing for community: England fans at the 2006 FIFA World Cup. Soccer Soc. 2008;9(3): 428–438. 10.1080/14660970802009056

[26] Panja T. V.A.R. check: in Premier League, almost no one is happy. The New York Times [Internet]. 2019 Nov 23 [updated 2019 Nov 25; cited 2020 Jun 14]. Available from https://www.nytimes.com/2019/11/23/sports/premier-league-var-mike-riley.html

[27] Weiss CH. Where politics and evaluation research meet. American Journal of Evaluation. 1993;14(1): 93–106. 10.1177/2F109821409301400119

[28] Kahneman D, Tversky A. Prospect Theory: an analysis of decision under risk. Econometrica. 1979;47(4): 263–291. 10.2307/1914185

[29] Blaszka M, Burch LM, Frederick EL, Clavio G, Walsh P. #WorldSeries: an empirical examination of a Twitter hashtag during a major sporting event. International Journal of Sport Communication. 2012;5(4): 435–453. 10.1123/ijsc.5.4.435

[30] Boehmer J. Does the game really change? How students consume mediated sports in the age of social media. Communication & Sport. 2016;4(4): 460–483. 10.1177/2167479515595500

[31] Lambov D, Pais S, Dias G. Merged Agreement Algorithms for Domain Independent Sentiment Analysis. Procedia Soc Behav Sci. 2011;27: 248–257. 10.1016/j.sbspro.2011.10.605

[32] Nadimpalli VK, Hasenbein JJ. When to challenge a call in tennis: A Markov decision process approach. J Quant Anal Sports. 2013;9(3): 229–238. 10.1515/jqas-2012-0051

[33] Anbarci N, Lee J, Ulker A. Win at all costs or lose grace- fully in high-stakes competition? Gender differences in professional tennis. J Sports Econom. 2014;17(4): 323–353. 10.1177/1527002514531788

[34] Carboch J, Vejvodova K, Suss V. Analysis of errors made by line umpires on ATP tournaments. Int J Perform Anal Sport. 2016;16(1): 264–275. 10.1080/24748668.2016.11868885

