# Supplementary material for: Video kills the sentiment—Exploring fans’ reception of the video assistant referee in the English premier league using Twitter data
Source: PLoS One. 2020 Dec 9;15(12):e0242728. doi: 10.1371/journal.pone.0242728 (PMC7725346; doi:10.1371/journal.pone.0242728)
Supplement: S6 Table — (DOCX) [file pone.0242728.s006.docx]

| **Metric** | **Formula** | **Notes** |
| --- | --- | --- |
| **Accuracy** | $\frac{true positives+true negatives}{total}$ | - *True positives*: observations correctly classified as member of a given category - *False positives*: observations incorrectly classified as member of a given category - *True negatives*: observations correctly classified as outside of a given category - *False negatives*: observation incorrectly not classified as member of a given category - *Total*: number of total observations |
| **Precision** | $\frac{true positives}{true positives+false positives}$ |  |
| **Recall** | $\frac{true positives}{true positives+false negatives}$ |  |
| **F-Score** | $2*\frac{Precision*Recall}{Precision+Recall}$ | *Precision* and *Recall* as described above |
| **Macro-averaged F-Score** | $\frac{F_{(+)}+F_{(-)}}{2}$ | *F_(+)_* is the *F-Score* for the detection of positive sentiment, *F_(-)_* the *F-Score* for the detection of negative sentiment. |
| **Krippendorff’s Alpha** | $1-\frac{observed disagreement}{expected disagreement}$ | See [20] and [22] for a detailed description for the application to discrete variables with three possible outcomes |
